# Personality, Attitudes, and Behaviors Predicting Perceived Benefit in Online Support Groups for Caregivers: Mixed Methods Study

**DOI:** 10.2196/36167

**Published:** 2022-08-18

**Authors:** Athena Milios, Ting Xiong, Karen McEwan, Patrick McGrath

**Affiliations:** 1 Department of Psychiatry Dalhousie University Halifax, NS Canada; 2 Centre for Research in Family Health Izaak Walton Killam Health Centre Halifax, NS Canada; 3 Institute of Health Policy, Management and Evaluation University of Toronto Toronto, ON Canada

**Keywords:** online support groups, personality, support group, online support, peer support, caregiver, caregiving, caring, mother, father, usage pattern, extraversion, neuroticism, neurotic, agreeable, benefit, eHealth, Canada, North America, parent, neurodevelopment disorder, attitude, online behavior, emotional support, perceived benefit

## Abstract

**Background:**

Online support groups (OSGs) are distance-delivered, easily accessible health interventions offering emotional, informational, and experience-based support and companionship or network support for caregivers managing chronic mental and physical health conditions.

**Objective:**

This study aimed to examine the relative contribution of extraversion, agreeableness, neuroticism, positive attitudes toward OSGs on social networking sites, and typical past OSG use patterns in predicting perceived OSG benefit in an OSG for parents and caregivers of children with neurodevelopmental disorders.

**Methods:**

A mixed methods, longitudinal design was used to collect data from 81 parents across Canada. Attitudes toward OSGs and typical OSG use patterns were assessed using the author-developed Attitudes Toward OSGs subscale (eg, “Online support groups are a place to get and give emotional support”) and Past Behaviors in OSGs subscale (eg, “How often would you typically comment on posts?”) administered at baseline—before OSG membership. The personality traits of extraversion, agreeableness, and neuroticism were assessed at baseline using the Ten-Item Personality Inventory. Perceived OSG benefit was assessed using the author-developed Perceived OSG Benefit scale (eg, “Overall, did you feel supported by other members in this group?”), administered 2 months after the initiation of OSG membership.

**Results:**

A hierarchical regression analysis found that extraversion was the only variable that significantly predicted perceived OSG benefit (*R*^2^=0.125; *P*<.001).

**Conclusions:**

The key suggestions for improving future OSGs were facilitating more in-depth, customized, and interactive content in OSGs.

## Introduction

### Background

Online Support Groups (OSGs), or internet support groups, are health-focused online communities accessible through specific websites or social networking sites (SNS). OSGs are increasing in use with the development of SNS, because they provide a means for caregivers to build social support networks [1]. Different channels of OSGs may influence OSG use and behaviors. For example, OSG users in closed groups via SNS may have higher levels of and more positive self-disclosure than those on public forums [2]. Overall, participating in OSGs can help caregivers promote their well-being [3], facilitate information exchange and interaction [4,5], reduce depressive symptoms, and improve the quality of life [6].

A major advantage of OSGs, among other distance-delivered health interventions, is that they are accessible to a wide range of individuals seeking support to manage their health and well-being, in addition to being very cost-efficient for the health care system [7]. OSGs facilitate access to psychological (emotional) support, informational support, and companionship [8]. OSGs have shown great promise; however, further investigation is needed to assess factors influencing participation and user well-being, as measured by self-assessed or perceived benefit from the group [9,10]. Many previous efforts in understanding OSGs do not use well-validated measures to predict or assess the characteristics of those who participate and benefit from OSGs [11]. Demographic variables such as socioeconomic status and gender do not provide a complete enough measure to explain why and how much people participate in and benefit from OSGs [1].

### Personality as a Predictor of OSG Use and Benefit

The personality traits found to be of the highest relevance in predicting OSG use and perceived benefit were extraversion [12], agreeableness [13-15], and neuroticism [14,16]. The openness and conscientiousness personality traits were not widely found to be predictors of OSG use or benefits. More specifically, Pornsakulvanich [13] found that of the Big Five personality traits, agreeableness positively predicted satisfaction (a construct closely related to perceived benefit) from online social support. Agreeableness is characterized by cooperativeness, generosity, sympathy, and altruism [17]. Agreeableness has been shown to predict SNS use; for example, it is related to the number of pages viewed by SNS users [14,15]. Moreover, SNS (eg, Facebook) users in general (not just OSG users) also tend to be more extroverted than nonusers [12,14]. Extraversion is characterized by sociability, assertiveness, and the ability to experience positive emotions [17,18]. Extroverts tend to belong to more Facebook support groups [19] and are more likely to use SNS to establish social connections and friendships [15,20]. Furthermore, Moore and McElroy [21] found that neuroticism was associated with SNS (Facebook) use. Neuroticism is associated with distrustfulness, sadness, anxiety, embarrassment, and poor stress tolerance [17,18,21].

### Attitudes as a Predictor of OSG Use and Benefit

Overall attitudes toward SNS (not just OSGs specifically) can predict how often people use social media to find social support [13]. Attitudes toward SNS can also predict satisfaction with perceived support from SNS [13].

### Behaviors as a Predictor of OSG Use and Benefit

Caregivers of patients with autism spectrum disorder who participate in OSGs have positive views regarding OSGs and high levels of support satisfaction from the groups [22]. Coulson [23] found that OSGs help provide patients with both informational and emotional support and that participation in OSGs benefits patients with inflammatory bowel disease by helping members to be more positive and improve their sense of well-being.

### Demographics as Predictors of OSG Use and Benefit

Choi and colleagues [1] examined 5 components related to OSG participation: (1) demographics, (2) reading behaviors, (3) posting behaviors, (4) perceived roles in OSGs, and (5) values sought from OSGs. They found the most sought values in OSGs to be emotional support, experience-based informational support, unconventional informational support, and medical fact–based informational support. Lurkers (ie, passive OSG participants) demonstrate less extroverted behavior than posters, which could mean that lurkers possess certain preexisting characteristics or traits that make them less likely to actively participate in OSGs compared to posters [24]. Lurkers are also generally less satisfied with their OSG experience and benefit less from OSGs than those who actively post [8,24,25].

### Objective and Hypotheses

The main objective of this study was to examine how (1) personality, (2) attitudes toward OSGs, and (3) typical behaviors in OSGs explain perceived OSG benefit. This objective was evaluated through an OSG we developed via SNS (ie, Facebook). More specifically, the goal was to determine the relative contribution of the personality traits of extraversion, agreeableness, and neuroticism, as well as attitudes toward OSGs and typical past OSG use behaviors, in predicting perceived OSG benefit (the outcome variable). To be able to predict OSG participation and the health benefits that typically ensue, a reliable and validated measure of potential predictors such as personality factors, attitudes toward OSGs, and OSG use is needed.

It was hypothesized that extraversion, agreeableness, and neuroticism would be significant predictors of perceived benefit from the current OSG. A positive attitude toward OSGs was also expected to predict perceived OSG benefit [13,26]. Finally, it was hypothesized that past typical OSG use would predict perceived benefit from the current OSG.

## Methods

### Participants

The 2 intervention arms of an internet-based, 3-arm randomized controlled trial (RCT) [27] for caregivers of children with neurodevelopmental disorders and challenging behaviors formed the sample of the study. The control arm of the RCT did not receive invitations for OSG. The participants were 81 parents and caregivers of children aged 4-14 years, who are diagnosed with a neurodevelopmental disability including autism spectrum disorder, attention-deficit/hyperactivity disorder, cerebral palsy, Down syndrome, epilepsy, fetal alcohol spectrum disorder, global developmental delay, intellectual disability, learning disability, and spina bifida. Participants were recruited nationally in Canada through service provider clinics and organizations, emails, social media campaigns, posters, and brochures. No extra incentive was provided for taking part in the study. Informed consent was obtained through a web-based consent form administered through the mystudies.ca website. Participants were informed about the length of the survey, the purpose of the study, the investigator team, and data storage place and plan, and their questions about the study were answered before giving consent to the study. Access to the participants’ data was restricted to the trained staff of the research team via institutional laptops or computers with antivirus software. The completion rate was 50.6% (81/160) of the eligible participants who received the emails containing the surveys.

### Procedure

This study used mixed methods—there were both quantitative and qualitative components. Upon completing the pre-OSG baseline survey (Multimedia Appendix 1), participants were invited to join the closed Facebook OSG corresponding to their arm of the RCT program (either coached or self-managed) and provided with a welcome message containing guidelines for group participation. The surveys of the study were not advertised externally due to the closed membership of the OSG. After 2 months of OSG membership, the Perceived OSG Benefit scale (Multimedia Appendix 2) was administered. Both surveys were completed through the REDCap software (REDCap Consortium) [28], and the surveys were closed, which means that invitation links were shared with participants via email. Filling in the survey was entirely voluntary, and choosing not to participate did not influence any treatment that the participants were entitled to in the RCT.

### OSG Content

The OSGs were both asynchronous web pages for text-based, peer-to-peer networking. There was a parent facilitator in each group welcoming new parents and encouraging positive discussion. This facilitator was also a parent of a child with a neurodevelopmental disorder and had experience using OSGs. The parent facilitator posted regularly to help members engage with module content and encourage them to practice the skills they were learning with their children; the overall purpose of each OSG was to connect parents, providing a platform for them to discuss specific topics and skills (eg, parenting strategies) as they were learning them, as well as to ask questions and share personal experiences. Parent facilitators received remuneration for their time. The OSG followed a relatively unstructured format—the role of the facilitator was simply to encourage discussion based on what members seemed interested in discussing, but there were no specific guidelines for the topics that had to be discussed as long as the issues were loosely related to the program and were appropriate based on the guidelines for group participation that they were given when they first joined the group.

### Scale Development

To solicit items for the surveys, a literature search of OSGs for illnesses was conducted. Based on this search, a pre-OSG survey containing Likert-style responses was developed, called Personality, Attitudes, and Behavior around Health Forums (see Multimedia Appendix 1), to assess personality and attitudes toward OSGs and typical OSG use behaviors. To measure perceived group benefit, a survey was developed and named Perceived OSG Benefit (Multimedia Appendix 2). Both surveys were assessed by a panel of 10 health psychology experts and a panel of 10 parent advisors to improve the clarity of the items, solicit additional scale items, and assess the face and content validity of the items in each scale. Each member gave their independent assessment of whether the items in each scale were interrelated and whether they were true measures of the construct each subscale was designed to assess. The experts also commented on the practicality of the tools. Feedback regarding ambiguous, repetitive, or undesirable items was provided. The feedback was used to create a revised draft. The use of reverse-coded items was minimized for clarity purposes, to avoid cognitive burden for participants, and to reduce respondent fatigue [29].

### Measures

#### Demographics and Past OSG Behaviors

The first section of the baseline survey asks about demographic variables (ie, age, gender, duration of illness, and length of previous experience with OSGs). The next 4 questions ask about the nature of their typical participation patterns in OSGs (eg, their frequency of posting, commenting, and reacting to and viewing posts) based on all previous OSGs the parents have joined. An example of the questions is “How often would you typically comment on posts?”

#### Personality

The Ten-Item Personality Inventory includes 10 questions assessing the Big Five personality traits [30]. It has shown good construct validity and consistency reliability in a range of samples [30]. This study used the subscales to assess extraversion, agreeableness, and neuroticism. The items were each scored on a scale from 1-7, and their average was subsequently calculated for each trait. In this study, the internal consistency was 0.77 for extraversion, 0.52 for agreeableness, and 0.43 for neuroticism.

#### OSG Attitudes

The OSG attitudes subscale contains 10 questions related to attitudes (ie, beliefs and expectations) toward OSGs, including motivations for why an individual may want to use an OSG. There are questions regarding attitudes toward the usefulness of OSGs in providing various types of social support: emotional support (ie, “Online support groups are a place to get and give emotional support.”), medical and health-related informational support, and experience-based informational support [1,8,13,31,32]; the perceived trustworthiness of OSGs [1,31]; its usefulness for hope [23,24]; connection and friendship [33]; perceived enjoyment [13]; reducing isolation; raising awareness surrounding chronic illness [33]; and the sense of community [31].

#### Perceived OSG Benefit Scale

The Perceived OSG Benefit scale assesses to what extent participants perceived benefit from the OSG. The first 9 questions were adapted from previous literature and are rated from 1 to 5 on a Likert-type scale, with higher scores indicating higher perceived benefits. They ask about the perceived benefits of the OSG regarding providing support [22,33] (ie, “Overall, did you feel supported by other members in this group?”); meeting participant needs [34,35]; solving problems [22,35]; increasing parental hope [23]; reducing caregiver distress [22]; increasing self-efficacy [22,36]; well-being [23]; sense of community [31]; and overall satisfaction [1]. Question 10 (open-ended and qualitative) asks if there is anything else participants would like to add about how the group helped (or did not help) them.

### Data Analysis

For the quantitative data, an a priori power analysis was performed using G*Power statistical software (version 3.1) [37]. The required sample size was calculated to be 80. Only individuals who completed all measures were included in the analyses. The psychometric properties of the 3 author-constructed scales (ie, OSG Attitudes, Past Behaviors, and Benefits) were assessed by internal consistency (ie, Cronbach *α* coefficient) and construct validity (ie, exploratory factor analyses).

A multiple hierarchical linear regression was performed to analyze the proportion of variance that the outcome variable was accounted for by each predictor (independent) variable. The 5 predictor variables were the total scores for extraversion, agreeableness, neuroticism, attitudes toward OSGs, and typical past OSG use behaviors; the outcome variable was perceived OSG benefit. The regression assumptions of (1) normally distributed residuals (the error terms must be normally distributed), (2) homoscedasticity (the variance of the errors must be roughly constant around the least-squares line), (3) linearity (the predictor variables must have a linear relationship with the outcome variables), (4) no multicollinearity, and (5) the absence of influential outliers were checked before running the regression to confirm that the data were suitable for a regression analysis.

The qualitative data were analyzed using an inductive thematic analysis by AM [38]. The main themes were extracted from each participant’s written (open-ended) response, following the steps outlined by Braun and Clarke [38]. In the first step, each response was read multiple times to get a general feel for the content. In the second step, the data were systematically reduced into smaller chunks by looking for emerging issues within each response. In the third step, preliminary themes were extracted. In the final step, the responses were all reviewed and compared among each other to develop and solidify common themes and subthemes [38,39].

### Ethics Approval

This study was approved by the Research Ethics Board at the IWK Health Centre (1023970). The study was performed in accordance with the guidelines and regulations from the Research Ethics Board of IWK Health Centre. In addition, informed consent was obtained from the participants, and the respondents were fully informed of the purpose and procedures of the study. They were also assured of the confidentiality of information.

## Results

### Descriptive Statistics

The descriptive statistics (Table 1) revealed some interesting findings: notably, the entire (81/81, 100%) sample was female; most (33/81, 41%) have had their child’s diagnosis for over 5 years; and roughly half (39/81, 48%) had been using OSGs for over 3 years, meaning that they were experienced OSG users.

**Table 1:** Demographic characteristics and online support group (OSG) use behaviors (N=81).

Demographic variable		Participant, n (%)
**Age of caregiver (years)**		
	18-29	5 (6)
	30-49	67 (83)
	50-64	8 (10)
	≥64	1 (1)
**Gender of caregiver**		
	Male	0 (0)
	Female	81 (100)
**Intervention group**		
	Coached	40 (49)
	Self-managed	41 (51)
**Time since child’s diagnosis (years)**		
	<1	19 (23)
	1-3	18 (22)
	3-5	11 (14)
	>5	33 (41)
**Length of time accessing OSGs (years)**		
	<1	21 (26)
	1-2	21 (26)
	3-4	18 (22)
	≥5	21 (26)
**Frequency of creating posts in OSGs**		
	Once a month or less	47 (58)
	Every other week	15 (18)
	Weekly	16 (20)
	Daily or almost daily	3 (4)
**Commenting frequency on posts**		
	Once a month or less	24 (30)
	Every other week	26 (32)
	Weekly	14 (17)
	Daily or almost daily	17 (21)
**Reacting (like, dislike, and love, etc) frequency to posts**		
	Once a month or less	18 (22)
	Every other week	11 (14)
	Weekly	21 (26)
	Daily or almost daily	31 (38)
**Viewing frequency of posts**		
	Once a month or less	2 (2)
	Every other week	3 (4)
	Weekly	14 (17)
	Daily or almost daily	62 (77)

### Psychometric Properties of Author-Constructed Scales

The internal consistency reliabilities of the Perceived OSG Benefit scale, the Attitudes Toward OSGs subscale, and Past Behaviors in OSGs subscale were Cronbach *α*=0.93, 0.78, and 0.82, respectively. Exploratory factor analyses were conducted to explore the factor structure and preliminarily assess the construct validity of the Attitudes Toward OSGs subscale and the Perceived OSG Benefit scale. Both scales revealed 1 principal component (construct), and each item had clear conceptual coherence with the main construct (latent variable) it was designed to measure (ie, attitudes toward OSGs and perceived OSG benefit). The items in the Attitudes Toward OSGs survey were part of a larger measure, “Personality, Attitudes, and Behaviors around Health Forums.” The eigenvalue was 4.0, and the principal factor extracted explained 39.8% of the variance in the latent variable (positive attitudes toward OSGs). Factor analyses of the Perceived OSG Benefit scale also led to the extraction of 1 main factor (construct), providing evidence that the construct of self-reported OSG benefit is in fact unidimensional, meaning all the items in the self-reported OSG benefit survey were designed to assess benefit derived from OSGs. The eigenvalue for the principal factor was 6.1; this factor explained 68.3% of the variance in the latent variable (perceived OSG benefit) and was therefore considered an excellent approximation of what this survey was designed to measure, supporting its construct validity.

### Regression Analysis

Before performing the regression analysis, the correlation matrix among all the variables was examined. The correlation between extraversion and perceived OSG benefit was the only significant correlation (at the 0.01 level, 2-tailed)—the Pearson correlation coefficient for these 2 variables was 0.35. After the regression analysis was performed, extraversion was found to be the only major predictor of self-reported OSG benefit (*R*^2^=0.125; *P*<.001). Even though the 5 predictor variables all together significantly predicted perceived OSG benefit (ie, the overall regression model was found to be significant; *P*=.02), extraversion was the only significant predictor of self-reported OSG benefit, explaining 12.5% of the variability in this outcome variable. The other 4 predictor variables combined only explained 3% of the variance (agreeableness: *P*=.36; neuroticism: *P*=.68; positive attitudes toward OSGs: *P*=.35; typical past OSG use pattern: *P*=.33).

Neither positive attitudes toward OSGs nor typical past OSG use patterns significantly predicted perceived benefit from the current OSG, meaning that neither of these hypotheses (hypothesis 2 and hypothesis 3) were supported.

### Qualitative Results

The last question on the Perceived OSG Benefit scale (“Do you have anything else to add about your experience using the group or how the group helped or didn’t help you? If so, could you provide some examples of how the group had an impact on you?”) was answered by 73 (90%) out of 81 participants; 65 of the responses were relevant to the question being asked. Table 2 displays the main themes extracted and the number of participants who considered the theme important or relevant. A list of suggestions was also compiled, based on the open-ended responses of improvements suggested by the parents and caregivers, which could be useful for creators or facilitators to improve health and well-being–related outcomes from future OSGs.

The main theme was the lack of engagement, activity, and interaction, particularly from the OSG moderators, due to the paucity of posts; they are more likely to be irrelevant and for the OSG to be dismissed by its members (n=27). Main suggestions for improving future health-related OSGs were (1) posting psychoeducational content directly related to the needs of the patients/caregivers in the OSG; (2) expanded, larger groups could be more helpful due to acquiring more resources and diversity of opinions; and (3) more in-depth, proactive discussion generated by the moderators.

**Table 2:** Main themes and suggestions for the benefits of future health-related online support groups (OSGs; n=65).

Themes and suggestions		Participant, n	Illustrative quotes
**Negative OSG themes**			
	The lack of engagement, activity, and interaction, particularly from the OSG moderators, due to the paucity of posts; they are more likely to be irrelevant and for the OSG to be dismissed by its members	27	“There just wasn’t a lot of conversation happening in my group. It was very small, and the content of the posts was largely composed of introductions.”
	The lack of awareness of the OSG, lack of ability to use the social networking sites on which the OSG was hosted (Facebook), or difficulty navigating the OSG’s page (lack of digital literacy)	6	“I’m not typically an online/Facebook user.” “I don’t really go on Facebook. I finally went on and signed up.”
	The lack of interpersonal connection and relevance of posts in the OSG; comments were found by some members to be superficial (lacking reflection or insight), general, and impersonal	6	“I found it hard to connect as I’m used to being on my own as a single parent. Most were in relationships and felt I couldn’t relate.” “Real connection is difficult to establish online.”
**Positive OSG themes**			
	Feeling connection, encouragement, positivity, and reassurance from the OSG	4	“It’s good to relate to other parents who are having some of the same experiences as I.”
	Felt welcome to share in the OSG and appreciated reading about situations or experiences that others shared for learning purposes	3	“Reading about others’ struggles or experiences is helpful as I don’t feel like it’s just my family that struggles. I also like that people have posted articles or videos that have been helpful and align with the skills we are learning.”
	The OSG was well moderated, and shared experiences, and invited feedback and conversation	2	“I do appreciate that it is well moderated.”
**Main suggestions for improving future health-related OSGs**			
	Posting psychoeducational content directly related to the needs of the patients/caregivers in the OSG	N/A^a^	“I think that it would be more interesting if the moderator posted content that directly relates to what we cover in our phone conversations.”
	Expanded, larger groups could be more helpful due to acquiring more resources and diversity of opinions	N/A	“I find the larger the group within a community (such as Calgary or Alberta Autism), the better. It allows for enough similarity of situation and enough diversity of thought.”
	More in-depth, proactive discussion generated by the moderators (eg, asking questions that patients/caregivers can respond to) to engage members and encourage their input to posts (patients/caregivers who are putting themselves out there by creating posts want to feel heard and empathized with)	N/A	“It could be a better source of support if there was more discussion generated by the moderators and more input from the participants.”
	More sharing of personal experiences, as well as sharing more complex, deep, and vulnerable posts	N/A	“I’d like to see more controversial comments. I found comments to be very one-note and fluffy. I like sharing/hearing personal stories, it would just be nice for them to go deeper. I also didn’t feel comfortable sharing in this type of setting. It made me question whether comments would be genuine and whether my opinions would be taken well. I didn’t feel the ‘opportunity cost’ was there in terms of energy it would require to share/feel my opinions were interpreted correctly vs. benefit I’d receive.”
	Making OSGs more targeted, so that there is a stronger “common denominator” among the members; mandating regular posting if patients/caregivers want to stay in the group to increase their motivation to engage	N/A	“I am part of other groups where parents with kids with the same diagnosis are together and those groups are FAR more supportive and informative.”
	Mandating regular posting if patients/caregivers want to stay in the group, in order to increase their motivation to engage.	N/A	“I don’t feel like we are all engaging in it enough because it’s voluntary. If everyone was told to post once a week about an experience or to comment it would be more interactive.”

^a^N/A: not applicable.

## Discussion

### Findings and Implications

This study used a longitudinal mixed design to investigate the predictors of perceived benefits from OSGs among 81 parents of children with neurodevelopmental disorders and challenging behaviors. For this study, we developed and evaluated the Perceived OSG Benefit scale. A secondary analysis generated experiences and suggestions for OSG designs.

Our study yielded 3 principal findings. First, extraversion significantly predicts self-reported OSG benefit in a sample of caregivers of children with neurodevelopmental disorders and challenging behaviors.

Second, suggestions for future health-related OSGs for caregivers were made. They include customizing psychoeducational content; expanding the size of the OSG; more in-depth, proactive discussion; and more sharing of personal experiences and complex, deep, and vulnerable experiences. The qualitative data helped us understand what worked and what did not work in the OSGs.

Lastly, the Perceived OSG Benefit scale created in this study was found to have excellent internal consistency reliability, face validity, and content validity (ie, accurately, dependably, and effectively measuring the full depth and breadth of the construct it was designed to measure), which means it can be used by future researchers to measure benefit from other health-related OSGs. It can be used in the planning, facilitation, and assessment of future OSGs, to help clinicians and other facilitators run these groups in a way that leads to the greatest improvement in health and wellness–related outcomes of the patients/caregivers in the group. Improving the facilitation of OSGs could help caregivers build socioemotional connections and reduce their stress levels and isolation.

### Limitations and Future Directions

The primary limitation of this study was that the caregivers participating were also completing a separate intervention, which may have confounded the assessment of perceived benefit from the OSGs. The membership of the OSGs in this study depended on the affiliated intervention; therefore, their experience with the intervention might have impacted their attitudes to and perceived satisfaction with the OSGs. Another limitation is that the author-developed surveys used in this study had not been previously validated, so their generalizability (external validity) to OSGs for other parent or caregiver populations has not yet been determined. Furthermore, the internal consistency of the agreeableness and neuroticism sections of the Ten-Item Personality Inventory was poor. Future research should examine whether these traits predict OSG benefit using a longer, more thorough assessment of the Big Five personality traits (with higher internal consistency reliability), such as the Big Five Personality Inventory (a 50-item measure) [17]. The sampling method and design of the study may limit the generalizability of our findings. First, this study only examined the OSGs on Facebook as an example, whereas the other OSG users on public forums or websites may show different behaviors and patterns. Second, the OSGs designed in the study may have empowered extroverts to benefit from the group, and they may be more likely to participate in the web-based study. Future studies should use more rigorous designs, such as randomized controlled trials, to test this effect.

Since the preliminary validation of both author-created surveys was successful, they may be used by future researchers and clinicians to identify who would be more likely to participate in and benefit from OSGs. These assessments could then guide clinicians (and other facilitators) in tailoring the design of their OSGs to the specific strengths and vulnerabilities of the members. A flexible, caregiver-centered facilitation of OSGs could be more effective in improving the health and wellness outcomes of those who have preexisting characteristics that predict that they are less likely to benefit from OSGs, such as being introverts. Future research could test various methods (eg, post recommendations and reminders) of engaging caregivers who are less likely to participate in and benefit from OSGs (eg, introverts). Future designs of OSGs should boost their positive effects in both introverts and extroverts and empower both “posters” and “lurkers” to receive high levels of social support, such as posting useful information via OSG moderators.

Future research should further test and validate these author-created surveys in other populations, both for caregivers and patients dealing with a range of different health conditions. More research is also needed to examine why and how extraversion predicts greater perceived benefit (but also actual benefit) from OSGs, as well as using a larger scale (such as the Big Five Personality Inventory) to more thoroughly assess extraversion, agreeableness, and neuroticism in relation to increased OSG benefit. Future studies could also expand on the qualitative findings of this study and test the 6 recommendations for improving health-related OSGs provided by these parents and caregivers.

### Conclusions

This study designed and evaluated the Perceived OSG Benefit scale and used a longitudinal design to examine the predictors of OSG benefits. We identified extraversion as a significant predictor of benefits from current OSG designs. Qualitative results yielded the current experiences and suggestions of OSG among parents of children with neurodevelopmental disorders, including customizing psychoeducation; expanding the size of the OSG; more in-depth, proactive discussion; and more sharing of personal experiences and complex, deep, and vulnerable experiences. This study lays a foundation for future studies that aim to study OSG benefits and customize OSG designs for parents of children with neurodevelopmental disorders.

## Appendix

Multimedia Appendix 1 Personality, Attitudes, and Behavior around Health Forums survey.

Multimedia Appendix 2 Perceived OSG Benefit scale. OSG: online support group.

## Abbreviations

OSG: online support group
RCT: randomized controlled trial
SNS: social networking sites

## Data Availability

Deidentified data sets used and analyzed during this study are available from the corresponding author on reasonable request with the permission of the Research Ethics Board of the Izaak Walton Killam (IWK) Health Centre.
