# Supplementary material for: Personality, Attitudes, and Behaviors Predicting Perceived Benefit in Online Support Groups for Caregivers: Mixed Methods Study
Source: JMIR Nurs. 2022 Aug 18;5(1):e36167. doi: 10.2196/36167 (PMC9437785; doi:10.2196/36167)
Supplement: Multimedia Appendix 1 [file nursing_v5i1e36167_app1.docx]

**Multimedia Appendix 1. Personality, Attitudes, and Behavior around Health Forums survey.**

| **Demographics and Typical Behaviors in Online Support Groups**  **What is your age?**  Under 18 years old  18-29 years old  30-49 years old  50-64 years old  65 years old or older  **Are you:**  Male (1)  Female (2)  Other (3)  **How long has it been since your child was diagnosed?**  Less than one year (1)  1-3 years (2)  3-5 years (3)  5-10 years (4)  More than 10 years (5)  **How long have you been accessing online support groups?**  Never accessed one before (1)  Less than a year (2)  1-2 years (3)  3-4 years (4)  5 or more years (5) |
| --- |
| The following questions refer to Online Support Groups that you have been a member of in the past. Please think carefully about your past experiences and try to give as accurate an estimation as possible.  **How often would you typically *post*?**  Never (1)  Once a month or less (2)  Every other week (3)  Weekly (4)  Daily or almost daily (5)  **How often would you typically *comment* on posts?**  Never (1)  Once a month or less (2)  Every other week (3)  Weekly (4)  Daily or almost daily (5)  **How often would you typically *react* to posts/comments (click like, dislike, love, etc.)?**  Never (1)  Once a month or less (2)  Every other week (3)  Weekly (4)  Daily or almost daily (5)  **How often would you typically *view* posts/comments?**  Never (1)  Once a month or less (2)  Every other week (3)  Weekly (4)  Daily or almost daily (5) |
| **Personality**  Please write a number next to each statement to indicate the extent to which you agree or disagree with that statement. You should rate the extent to which the pair of traits applies to you, even if one characteristic applies more strongly than the other.  Disagree strongly (1)  Disagree moderately (2)  Disagree a little (3)  Neither agree nor disagree (4)  Agree a little (5)  Agree moderately (6)  Agree strongly (7)  1. _____ Extraverted, enthusiastic.  2. _____ Critical, quarrelsome.  3. _____ Dependable, self-disciplined.  4. _____ Anxious, easily upset.  5. _____ Open to new experiences, complex.  6. _____ Reserved, quiet.  7. _____ Sympathetic, warm.  8. _____ Disorganized, careless.  9. _____ Calm, emotionally stable.  10. ____ Conventional, uncreative. |
| **Attitudes Toward OSGs**  Below are a number of statements regarding online support groups. Please read each statement carefully and indicate to what extent you agree or disagree with each. We are interested in your honest opinion, whether positive or negative. Thank you very much, we really appreciate your help. |
| **1. Online support groups are a place to get and give *emotional support.***  Strongly disagree (1)  Disagree (2)  Neutral (3)  Agree (4)  Strongly agree (5) |
| **2. Online support groups are a place to find new information about my child’s neurodiversity.**  Strongly disagree (1)  Disagree (2)  Neutral (3)  Agree (4)  Strongly agree (5) |
| **3. Online support groups are a place to share ideas/personal experiences/struggles about my child’s neurodiversity and to get feedback from people who have similar issues.**  Strongly disagree (1)  Disagree (2)  Neutral (3)  Agree (4)  Strongly agree (5) |
| **4. Online support group content posted by other members is unreliable.**  Strongly disagree (5)  Disagree (4)  Neutral (3)  Agree (2)  Strongly agree (1) |
| **5. Online support groups help members to take a more hopeful attitude towards life.**  Strongly disagree (1)  Disagree (2)  Neutral (3)  Agree (4)  Strongly agree (5) |
| **6. Online support groups are a place to connect with people like me and make new friends.**  Strongly disagree (1)  Disagree (2)  Neutral (3)  Agree (4)  Strongly agree (5) |
| **7. Participating in online support groups is enjoyable.**  Strongly disagree (1)  Disagree (2)  Neutral (3)  Agree (4)  Strongly agree (5) |
| **8. Participating in online support groups helps people to feel less isolated in their situation.**  Strongly disagree (1)  Disagree (2)  Neutral (3)  Agree (4)  Strongly agree (5) |
| **9. Online support groups are a place to raise awareness about issues relating to living with an ongoing health condition.**  Strongly disagree (1)  Disagree (2)  Neutral (3)  Agree (4)  Strongly agree (5) |
| **10. Online support groups provide a sense of community.**  Strongly disagree (1)  Disagree (2)  Neutral (3)  Agree (4)  Strongly agree (5) |
