# Supplementary material for: Personality, Attitudes, and Behaviors Predicting Perceived Benefit in Online Support Groups for Caregivers: Mixed Methods Study
Source: JMIR Nurs. 2022 Aug 18;5(1):e36167. doi: 10.2196/36167 (PMC9437785; doi:10.2196/36167)
Supplement: Multimedia Appendix 2 [file nursing_v5i1e36167_app2.docx]

**Multimedia Appendix 2. Perceived online support group (OSG) Benefit scale.**

The following questions refer to the Strongest Families “Parent-to-Parent Connections” group that you have been a member of. We would like to know how this group is doing and how it helped (or didn’t help) you. It should take you about 5 minutes. Please read each statement carefully and indicate to what extent you agree or disagree with each. We are interested in your honest opinion, whether positive or negative. Thank you very much, we really appreciate your help!

| **1. Overall, did you feel supported by other members in this group?**  No, there was a complete lack of support (1)  A little supported (2)  Some support (3)  Substantial support (4)  Yes, I felt fully supported (5) |
| --- |
| **2. To what extent has this group met your support needs?**  None of my needs have been met (1)  Only a few of my needs have been met (2)  Some of my needs have been met (3)  Most of my needs have been met (4)  Almost all of my needs have been met (5) |
| **3. Has the support you have received from this group helped you to deal more effectively with your child’s health challenges?**  No, definitely not (1)  No, not really (2)  Neutral (3)  Yes, generally (4)  Yes, definitely (5) |
| **4. Overall, has this group helped you to feel more hopeful for the future?**  No, definitely not (1)  No, not really (2)  Neutral (3)  Yes, generally (4)  Yes, definitely (5) |
| **5. Has this group reduced your day-to-day stress levels?**  No, definitely not (1)  No, not really (2)  Neutral (3)  Yes, generally (4)  Yes, definitely (5) |
| **6. Has this group improved your *confidence* in your ability to manage your child’s behavior?**  No, definitely not (1)  No, not really (2)  Neutral (3)  Yes, generally (4)  Yes, definitely (5) |
| **7. Has this group improved your general sense of *wellbeing*?**  No, definitely not (1)  No, not really (2)  Neutral (3)  Yes, generally (4)  Yes, definitely (5) |
| **8. Has this group made you feel part of a community?**  No, definitely not (1)  No, not really (2)  Neutral (3)  Yes, generally (4)  Yes, definitely (5) |
| **9. In general, how satisfied are you with this online support group?**  Very dissatisfied (1)  Dissatisfied (2)  Neither satisfied nor dissatisfied (3)  Satisfied (4)  Very satisfied (5) |
| **10. Do you have anything else to add about your experience using the group or how the group helped or didn’t help you? If so, could you provide some examples of how the group had an impact on you?** |
